# What’s Wrong with the Murals at the Mogao Grottoes: A Near-Infrared Hyperspectral Imaging Method

**DOI:** 10.1038/srep14371

**Published:** 2015-09-23

**Authors:** Meijun Sun, Dong Zhang, Zheng Wang, Jinchang Ren, Bolong Chai, Jizhou Sun

**Affiliations:** 1 School of Computer Science, Tianjin University, Tianjin, China; 2 School of Computer Software, Tianjin University, Tianjin, China; 3 Centre for excellence in Signal and Image Processing, University of Strathclyde, Glasgow, UK; 4 Dunhuang Academy, Gansu, China

## Abstract

Although a significant amount of work has been performed to preserve the ancient murals in the Mogao Grottoes by Dunhuang Cultural Research, non-contact methods need to be developed to effectively evaluate the degree of flaking of the murals. In this study, we propose to evaluate the flaking by automatically analyzing hyperspectral images that were scanned at the site. Murals with various degrees of flaking were scanned in the 126th cave using a near-infrared (NIR) hyperspectral camera with a spectral range of approximately 900 to 1700 nm. The regions of interest (ROIs) of the murals were manually labeled and grouped into four levels: normal, slight, moderate, and severe. The average spectral data from each ROI and its group label were used to train our classification model. To predict the degree of flaking, we adopted four algorithms: deep belief networks (DBNs), partial least squares regression (PLSR), principal component analysis with a support vector machine (PCA + SVM) and principal component analysis with an artificial neural network (PCA + ANN). The experimental results show the effectiveness of our method. In particular, better results are obtained using DBNs when the training data contain a significant amount of striping noise.

The Mogao Grottoes in Dunhuang, Northwestern China, are famous for their ancient murals and statues and are considered to be one of the most valuable cultural heritage sites in the world. People began to carve the caves in 336 AD and continued until the Yuan Dynasty (1271–1368 AD). Currently, 735 caves have been identified, which include approximately 45,000 square meters of frescoes and 2,415 painted clay sculptures. The murals are extensive and invaluable for the scale and richness of their content as well as their artistry. As shown in Fig. 1, the subjects of these murals are often Buddhist, and many of the caves are completely painted on the walls and ceiling with a seated Buddha or Flying Chinese Apsaras.

In the Mogao Grottoes, murals are painted on the rock surface, which are conglomerates. The painting layer consists of three sub-layers: a plaster layer, a white lime layer and a pigment layer that includes a mixture of azurite, minium and malachite. To improve the adherence of the pigment, animal-based glue was adopted. However, these precious murals are suffering from different types of degradation due to exposure to air and light and to human factors over the long time period. More recent research has shown that mural damage is primarily caused by the subflorescence of soluble salts along with water infiltration and migration. The changing humidity in the grottoes makes the plaster layer repeatedly expand and contract, resulting in a looser plaster layer as well as cracking and warping in the pigment layer.

Typical signs of degradation include flaking, net cracking, detachment, disruption and paint loss. In Fig. 2, we show an example of these degradations. Flaking refers to cracks in the pigment layer and results in warped fragments. Flaking occurs in more than 20% of the degraded area, and most of it is severe. Flaking is also regarded as an early stage of peeling, exfoliation, and delamination. As described elsewhere^1^,^2^,^3^,^4^,^5^, flaking murals are salvaged by reattaching the flakes using a polyvinyl acetate emulsion (PVA). This method was first put into practice in the 1950s to 1960s in the 94^th^ and the 108^th^ caves. A hybrid acrylic and silicone acrylic emulsion has been applied to flaking murals in the 23^rd^ and the 217^th^ caves during the past decade. A gelatin solution has been used in the 85^th^ and the 98^th^ caves. However, there is still a need for a more effective method of monitoring and assessing the level of flaking than human vision to evaluate these protection and reinforcement techniques. This will also help researchers determine if the protection is still working or is causing renewed damages. In this study, we analyze the flaking on murals in the Mogao Grottoes using NIR hyperspectral images of the murals and machine learning algorithms. This non-destructive technique shows great potential for assessing the state of the murals.

Hyperspectral imaging was first used to monitor cultural heritage in the mid-1990s, where the initial experiments were performed on the Parma Cathedral, Italy^6^. Later experiments exploited the imaging fluorescence LIDAR technique for the acquisition of hyperspectral fluorescence images of several monuments^7^,^8^,^9^. Some studies have focused on the detection and characterization of different lithotypes^6^,^10^ and protective treatments^11^,^12^,^13^. In addition, a few applications of imaging spectrometry in cultural heritage frameworks have been introduced in the literature. In particular, Buck *et al.*^14^ presented the results of the sub-pixel capability of detecting obsidian and pottery artifacts scattered on the bare soil surface of a site in the western USA. Palombi *et al.* elucidated the past interventions to conserve the Colosseum in Rome using hyperspectral fluorescence LIDAR imaging^15^. Hallstrom *et al.* used hyperspectral technology to document the restoration of soiled and biodeteriorated facades of the Colosseum in Rome^16^. These multispectral and hyperspectral imaging techniques are ideally suited for the examination of historical murals because they are non-contact and generally non-destructive.

## Results

### Spectral profiles

**Figure 3:**
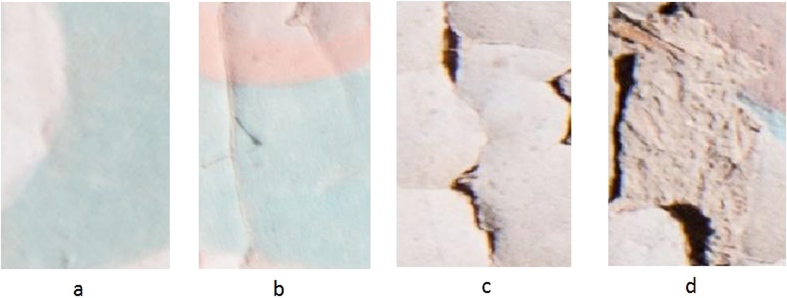
Different levels of flaking on the murals of Mogao Grotto No. 126. (**a**) A normal mural, (**b**) slight flaking, (**c**) moderate flaking, and (**d**) severe flaking.

There are different amounts of flaking areas in the mural images. To build a mechanism to detect flaking, we defined four levels based on its external form as shown in Fig. 3. As observed, (a) shows a normal mural, (b) shows slight flaking, (c) shows moderate flaking and (d) shows severe flaking. For the purpose of easy computation, values of these 4 levels were set to 0.0, 0.3, 0.6, and 1.0, which represent the four levels of damage from none (a normal mural) to severe, respectively. The reason that the four levels of flaking are unequally “spaced” is suggested by domain experts from the researchers of Dunhuang Academy. This will enable us to provide more divisions for fine detailed condition assessment from the beginning of flaking.

**Figure 4:**
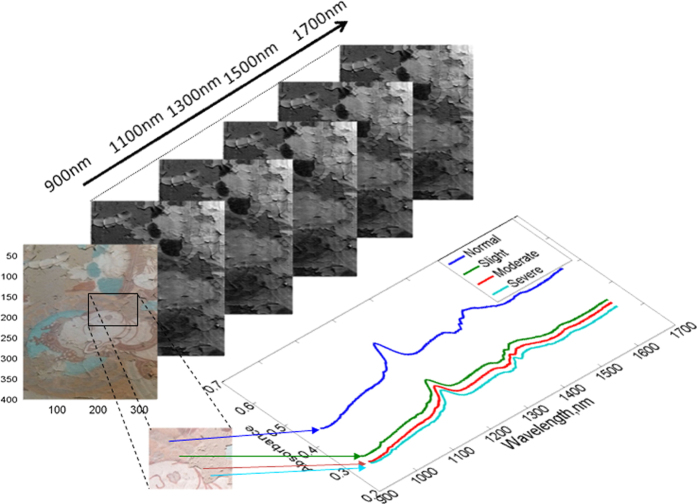
A hyperspectral image of the mural and its spectral curves for different levels of flaking (four levels are defined in this study as normal, slight, moderate and severe).

A hyperspectral image of the mural, shown in Fig. 4, was obtained using the NIR hyperspectral imaging system with wavelengths between 900 nm and 1700 nm. Normally, there are various levels of flaking on a mural. If a region of an image with flaking is selected, we can find some regions that are normal, some that are severe and some with flakes that have fallen off of the wall. In this study, for the defined four levels of flaking that varies from normal, slight, moderate to severe, their spectral profiles were extracted from the corresponding regions of the mural. As shown in Fig. 4, the spectral profiles have a similar trend, yet they are different in terms of the reflectance response in the spectral domain. The reflectivity of the normal region is the highest and that of the severe region is the lowest; the slight and moderate regions are intermediate but closer to the severe region. The significant difference in the spectra for the various degrees of flaking is important for predicting the degree of flaking.

### Prediction of the level of flaking

From each hyperspectral image, we manually selected 40 areas as ROIs according to their associated levels of flaking. As a result, each level of flaking is represented by ten different ROIs in each hyperspectral image. A total of 80 ROIs containing all four levels of flaking were collected from two hyperspectral images. In each ROI, there are approximately 150–200 pixels, and each pixel contains a data vector that represents its spectral profile (curve). From each ROI, 150 pixels are used to determine the spectral features of the ROI for prediction of the associated level of flaking. Eventually, we have 12,000 sample pixels for the four different degrees of flaking.

In this study, we repeated the training and prediction process ten times for each model and used the average results to evaluate the algorithm’s performance. In each repetition, 80% of the pixels for each flaking level were randomly selected from the 3000 sample points for the training data set, and the other 20% pixels were regarded as test data. To evaluate the models, the R-squared coefficient of determination and the root mean square error (RMSE) are used to show how well the predictions fit the observed data.


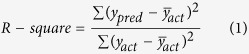



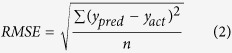


where *n* is the total number of sample points for predicting, *y*_*act*_ is the actual value, *y*_*pred*_ is the predicted value of the degree of flaking, as calculated using the model, and 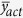 is the average of the actual values. All of the computations and multivariate data analyses were performed using Matlab.

The data analysis methods used in this study are DBNs, PLSR, PCA + SVM, and PCA + ANN. All the four algorithms were applied to the hyperspectral dataset which including 12000 pixels with 216 dimension data in wavebands space. First, the prediction method was used to create linear models that use the spectral data (X matrix) and the value of one of the flaking parameters obtained from the predefined values (Y matrix).

**Figure 5:**
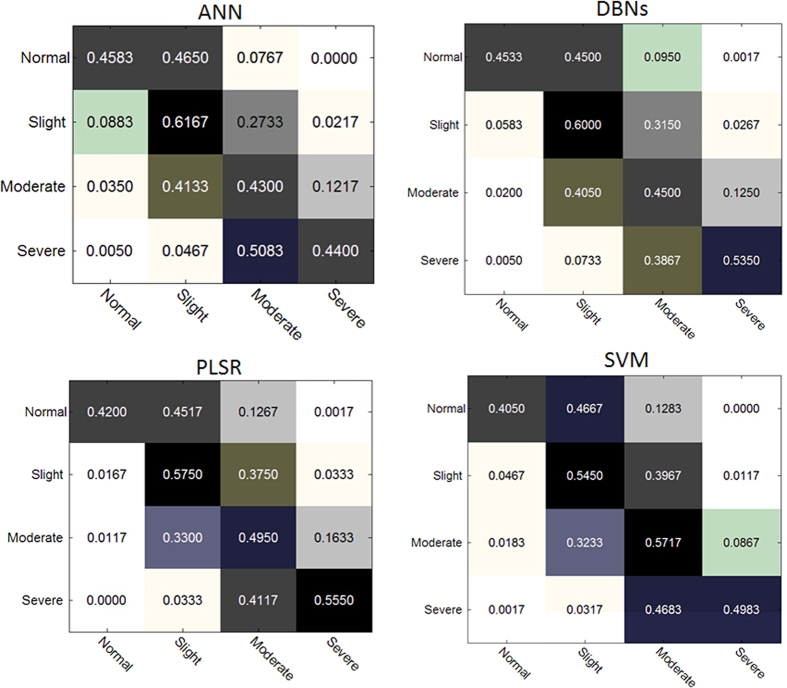
Confusion matrix of the prediction results to show different performance of these approaches in identifying various levels of flaking, where the DBNs method show better average performance than others.

As shown in Table 1, the DBN-based model performed better than the others. In addition, compared to conventional machine learning algorithms such as SVM and PLSR, whose results rely very much on the significance of the selected features, the DBN-based method does not need a strict selection of features. To further compare the performance of the four approaches in Table 1, the confusion matrix from them in identifying different levels of flaking are given in Fig. 5. As can be seen, both ANN and DBNs produce the best results in identifying normal and slight flaking cases, though ANN seems slightly better than DBNs. For the moderate and severe levels of flaking, the best results are generated by using SVM and PLSR, respectively. However, overall the DBNs outperform all three other approaches in this context.

To further improve on these results, a series of studies will be designed that include reducing the amount of striping noise in the hyperspectral data^17^, selecting the most discriminative band of the spectrum band, tuning the DBN parameters and considering larger sets of data. In addition, as these approaches perform differently in identifying various flaking levels, there is a potential to fuse them together for more improved data analysis. This will be further investigated in the future.

### Visual representation of flaking

**Figure 6:**
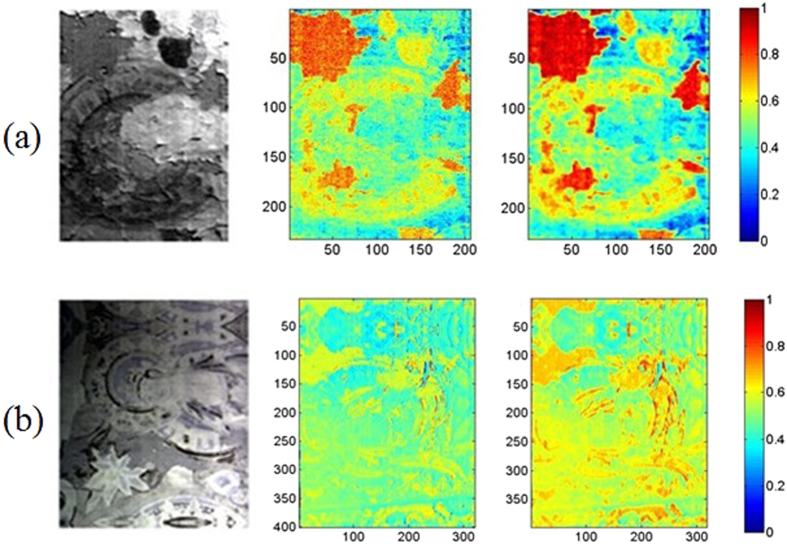
Distribution maps of the detected degree of flaking on real murals. The first column contains images of samples constructed by concatenating three spectral sub-images taken at 950 nm, 1200 nm and 1300 nm; the second column contains distribution maps showing the levels of flaking predicted using the DBN-based model; the third column shows the results of processing using a mean filter to highlight the worst areas.

With the trained DBN-based models, the flaking level can be visualized by predicting each pixel in the NIR hyperspectral image. The DBN-based model was selected as the predicting model because it outperformed all three of the other models. Figure 6 shows distribution maps of the degree of flaking on the murals. The images in the first column show the tested samples, the second column contains distribution maps of the predicted flaking values, and the third column contains mean filter-processed distribution maps that highlight the areas with the most damage. As expected, the level of flaking is nearly consistent with the level determined using human vision. The areas with flaking values between 0.05 and 0.3 are at risk of flaking, which provides a useful suggestion for protecting the murals at the Mogao Grottoes.

## Discussion

**Figure 7:**
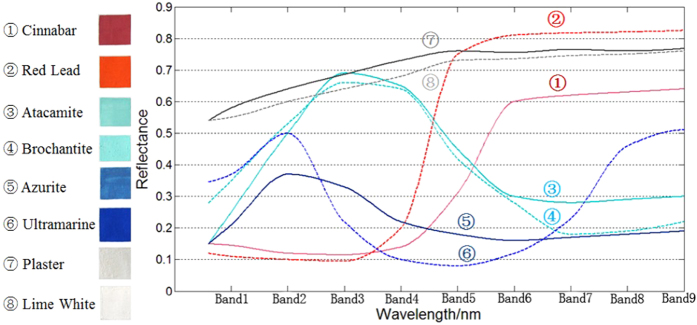
Different color pigments have different hyperspectral profiles due to the differences between their chemical components.

In general, the murals of the Dunhuang Mogao Grottoes contain pigment-based natural mineral pigments. Different color pigments have different chemical compositions and different sources^2^,^5^,^6^. As a result, their spectral profiles vary and differ from each other. This has been clearly shown in Fig. 7, where the spectral profiles from 8 color pigments are compared to show their differences.

The two images in our experiment are composed of several colors, including red, white, and cyan-blue. The main component of the red pigment is cinnabar, the white pigment is made from lime white, and the cyan-blue pigment is made from azurite. There are well-defined signatures with notable absorption bands that can be observed throughout the spectrum for all of the samples. The reflectivity intensities above 1180 nm are clearly high in these particular ranges. However, at 1180 nm, the reflectivity started to decrease sharply, a trend that continued to 1220 nm. Cinnabar, lime white and azurite’s main components are mercuric sulfide, calcium carbonate and azurite, respectively. The most prominent reflectivity bands occurring in the NIR region are related to overtones and combinations of the fundamental vibrations of O–H, C-O and C-H functional groups^18^. However, this is actually advantageous because the reflectivity bands that have sufficient intensity to be observed in the NIR region arise primarily from functional groups that have a hydrogen atom attached to an oxygen, carbon or nitrogen atom, which are common groups in the major constituents of compounds such as calcium carbonate and azurite. Additionally, bands in the 1400 nm region are ascribed to combinations of the bands for Ca, C-O and Cu vibrations^6^,^10^,^11^.

As a non-destructive technique for analyzing and assessing the components of materials, hyperspectral imaging shows a great deal of potential in many applications^19^,^20^,^21^,^22^. We have presented a quantitative approach to evaluating and visualizing the level of flaking damage to murals at the Mogao Grottoes in Dunhuang. Our promising results indicate that a combination of the NIR hyperspectral technique and a deep belief network is valuable. In addition to assessing the overall degree of damage caused by flaking on murals, hyperspectral imaging has the additional merit of allowing the distribution of the damage to be visualized.

In conclusion, the proposed hyperspectral method has been proven to capture the physical changes that will allow us to rank damage levels and predict mural damage prior to its occurrence. Further directions of this study include real-time monitoring, evaluating protection methods and early warnings of mural damage, which are considerably valuable for the protection of cultural heritage.

## Methods

### The Hyperspectral Imaging System

**Figure 8:**
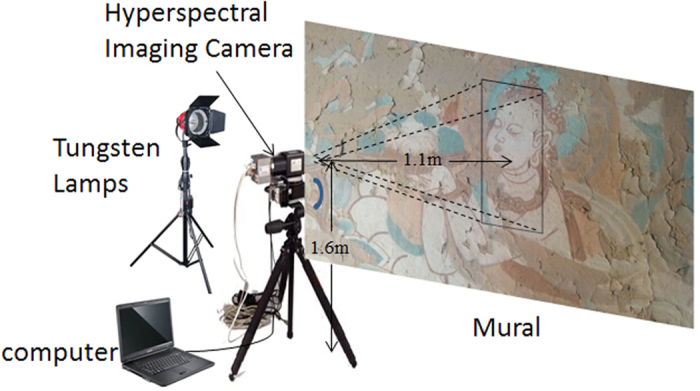
The hyperspectral imaging system.

A near-infrared hyperspectral imaging acquisition system was set up on site and used to collect hyperspectral data from the murals at the Mogao Grottoes in Dunhuang. As shown in Fig. 8, the system consisted of hardware and software components. The hardware consisted of a near-infrared spectral camera, a mobile platform, a computer, and two tungsten lamps that functioned as light sources for illumination. Computer software was used to control the platform’s speed and the exposure time. The spectral camera was fixed to the mobile platform 1.6 m above the ground and 1.1 m from the mural.

The NIR hyperspectral imaging system used in this study was a line scanning configuration that records each line of an image in one time step. The hyperspectral camera’s resolution was 320 × 400 pixels and its spectral range was from 882 nm to 1719 nm, with a spectral increment of approximately 3.3 nm, producing a total of 256 bands.

### Mural Data Acquisition and Calibration

Two historical murals from Dunhuang Mogao Grottoes No. 65 and 126, which date to the Tang Dynasty (618–907 AD), were used in this study. The major disease of these murals was flaking. Some sections of the murals were normal, but other sections exhibited flaking, which can significantly harm the murals. The NIR hyperspectral imaging system was used to acquire hyperspectral images of the murals. Spatial information was obtained in two dimensions (x, y) and spectral information was obtained in a third dimension (z); therefore, a three-dimensional (x, y, spectral) hyperspectral image was recorded.

Once the two murals had been scanned, the hyperspectral images (*R*_*0*_) were stored in a band interleaved by line (BIL) format. As a 3-dimensional image, the acquired hyperspectral image included both spatial and spectral information. To correct the effects of the light sources, R_0_ was corrected using a dark reference and a white reference as follows:


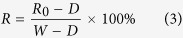


where *D* and *W* are respectively the dark current noise images obtained from the raw and white reference images, and *R* the calibrated image. The dark current noise image (approximately 0%) was acquired by recording a spectral image when the light was off and camera lens was completely covered with a black cap, and the standard white reference image was obtained by acquiring a spectral image from a high reflectance white calibration tile (approximately 99.99%).

To reduce the effect of noise, only wavelengths between 948 and 1654 nm with 216 spectral wavebands were used. To improve the signal to noise ratio (SNR), the minimum noise fraction (MNF) was used in this 216 dimension spectrum.

### The Solution Framework

**Figure 9:**
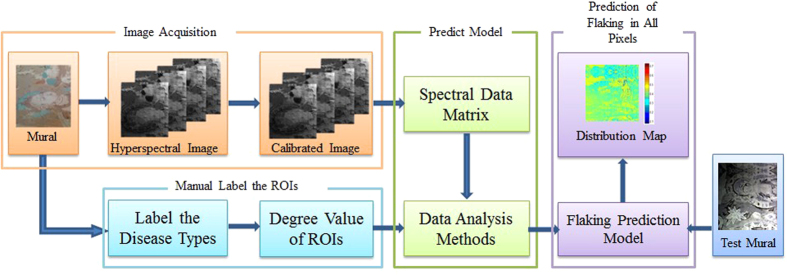
A flowchart of the method for assessing the degree of flaking on murals at the Mogao Grottoes in Dunhuang using near-infrared hyperspectral imaging.

Figure 9 illustrates the framework of the proposed solution. With the scanned hyperspectral image data and labeled dataset as input, the procedure was followed for four data analysis methods. Finally, visualizations of the pixel-based degrees of flaking were presented. First, the hyperspectral imaging system was used to acquire images of the two murals, and then, we manually labeled the ROIs. Second, four predictive models were used to analyze the data and to predict the degree of flaking from the images of the murals. Finally, the amount of flaking was predicted for each pixel to create a distribution map of flaking in all of the test images.

### Data Analysis Methods

The data were analyzed to predict the degree of flaking on the murals using the spectral profile^18^,^23^. In this study, we used four predictive models, which included deep belief networks (DBNs), partial least squares regression^24^ (PLSR), principal component analysis with a support vector machine^25^ (PCA + SVM), and principal component analysis with an artificial neural network (PCA + ANN).

DBNs have been successfully applied to a number of tasks that have benefitted from the recent discovery of this efficient learning technique^26^,^27^,^28^,^29^. DBNs learn a multi-layer generative model from data, and the features discovered by this model are then used to initialize a feed-forward neural network, which is fine-tuned using back propagation. In this study, we used a DBN-initialized neural network to predict the degree of flaking. In our experiments, for the two hidden layers, the hidden units were configured as 200 and 150, respectively. We pre-trained the weights of the DBNs layer by layer, from the first hidden layer to the outermost layer; the learning rate was set to 0.01 for 100 epochs, and the momentum was set to 0.4.

PLSR^18^,^30^,^31^,^32^ was used to create linear predictive models based on the spectral data (denoted as the X matrix) and the manually labeled degrees of flaking (denoted as the Y matrix). PLSR is a quantitative spectral decomposition technique that is used to optimize the covariance between Y and linear combinations of X by simultaneously decomposing the spectral and quality data.

PCA^33^,^34^ was performed on the entire spectral data set (the X matrix) to identify the spectral outliers. PCA is based on identifying the most important directions of variability in a multivariate data space (the X matrix) to identify the primary phenomena in the data set. The results of the PCA are then fed to an SVM^35^,^36^,^37^,^38^ and ANN^19^,^39^, in which the extracted principal components are used for modeling and prediction.

## Additional Information

**How to cite this article**: Sun, M. *et al.* What’s Wrong with the Murals at the Mogao Grottoes: A Near-Infrared Hyperspectral Imaging Method. *Sci. Rep.* **5**, 14371; doi: 10.1038/srep14371 (2015).

## Figures and Tables

**Figure 1:**
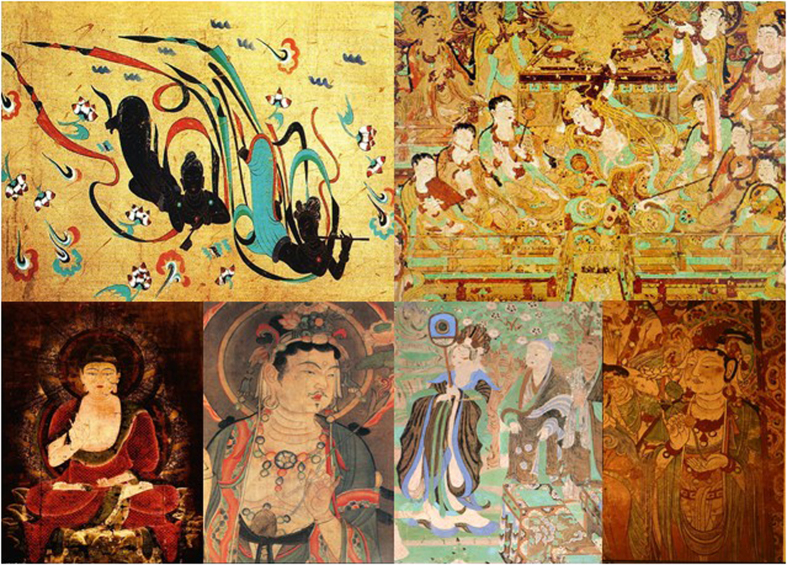
Examples of murals at the Mogao Grottoes (We are grateful to Bolong Chai for taking these photographs).

**Figure 2:**
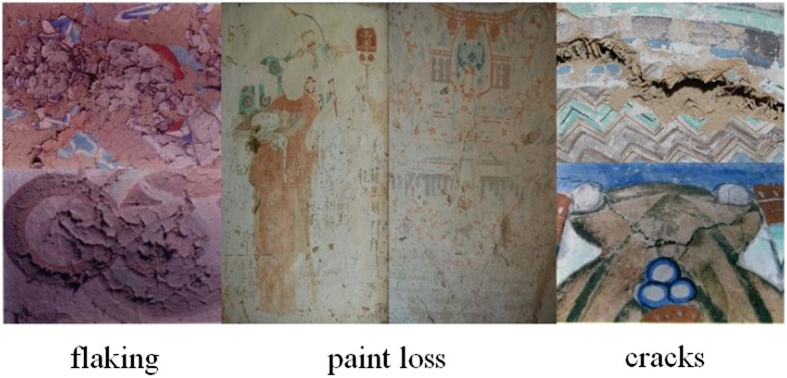
Some examples of diseases on the murals (We are grateful to Bolong Chai for taking these photographs).

**Table 1:** The four models used to predict the degree of flaking when the training and test data sets are equally large using the full spectral range (216 wavelengths).

	**R-square**	**RMSE**
DBNs	**0.5409**	**0.2482**
PLSR	0.5045	0.2605
PCA + SVM	0.4918	0.2629
PCA + ANN	0.4685	0.2698
